# Synergistic Induction of Oxidative and Endoplasmic Reticulum Stress by Tempol and ML210 Combination Therapy in B16F10 Melanoma Cells

**DOI:** 10.3390/ijms27062675

**Published:** 2026-03-14

**Authors:** Ebru Çelik, Percin Pazarci, Ömer Kokaçya, Halil Mahir Kaplan

**Affiliations:** 1 Department of Dermatology, Siirt Training and Research Hospital, Siirt 56000, Türkiye; 2 Department of Medical Biology, Faculty of Medicine, Cukurova University, Adana 01330, Türkiye; percinpazarci@gmail.com; 3 Department of Plastic, Reconstructive and Aesthetic Surgery, Faculty of Medicine, Cukurova University, Adana 01330, Türkiye; okokacya@cu.edu.tr; 4 Deparment of Pharmacology, Faculty of Medicine, Cukurova University, Adana 01330, Türkiye; mkaplan@cu.edu.tr

**Keywords:** melanoma, Tempol, ML210, oxidative stress, ER stress, ferroptosis

## Abstract

Given the challenges in treating metastatic melanomas, there is a growing need for novel and effective therapeutic strategies. This study aimed to understand molecular mechanisms underlying synergistic effects of a Tempol and ML210 combination in B16F10 murine melanoma cells and to evaluate its therapeutic potential. We hypothesized that this combination would synergistically induce cell death by increasing oxidative stress and triggering ER stress. B16F10 melanoma cells were treated with Tempol and ML210 alone or in combination for 48 h. Cell viability was determined using MTT assay. Oxidative stress was evaluated by measuring Total Antioxidant Status (TAS), Total Oxidant Status (TOS), and intracellular H_2_O_2_ levels. Apoptotic markers (caspase-3, Bax, Bcl-2) and ER stress proteins (GRP78, GADD153, IRE1α, ATF6) were quantified by ELISA. Combination treatment significantly inhibited cell proliferation compared to monotherapies. Molecular analyses revealed that combination caused depletion of TAS and increase in TOS and intracellular H_2_O_2_ levels. Furthermore, combination treatment synergistically upregulated ER stress markers and pro-apoptotic proteins while significantly suppressing anti-apoptotic Bcl-2 expression. In conclusion, the combination of Tempol and ML210 synergistically induces cell death in B16F10 melanoma cells by disrupting redox balance and activating ER stress-mediated apoptosis. These findings suggest a potential strategy for melanoma treatment that warrants further in vivo investigation.

## 1. Introduction

Melanoma, derived from melanocytes, is an aggressive skin cancer with the ability to metastasize rapidly. The rising incidence of melanoma in recent years makes early diagnosis and the development of effective treatment strategies imperative. Treating advanced-stage melanomas poses significant challenges due to tumor heterogeneity, drug resistance mechanisms, and a limited number of treatment options [1]. Therefore, there is a need for innovative therapeutic approaches that can enhance the effectiveness of existing treatments and overcome resistance mechanisms [2].

Furthermore, melanoma cells exhibit a unique redox biology that can be exploited therapeutically. Due to intrinsic factors such as deregulated melanin synthesis and high metabolic rates, melanoma cells typically maintain elevated basal levels of reactive oxygen species (ROS) compared to normal melanocytes [3,4,5]. To survive this continuous oxidative pressure, these tumor cells become highly dependent on robust antioxidant defense systems, particularly the glutathione-dependent pathways [6]. This phenomenon, often termed ‘redox addiction’, presents a distinct vulnerability [7,8]. Interventions that simultaneously inhibit essential antioxidant nodes and exogenously overload the system with ROS can preferentially push melanoma cells beyond their toxicity threshold [9]. In this context, exploiting this specific biochemical Achilles’ heel provides a strong rationale for combined redox-directed therapies.

Oxidative stress and endoplasmic reticulum (ER) stress are cellular stress responses that play critical roles in cancer cell survival, growth, and death [10]. The phenomenon of oxidative stress results from a disparity between the excessive production of reactive oxygen species (ROS) and the organism’s ability to neutralize these intermediates via antioxidant mechanisms [11]. High ROS levels can lead to cellular damage such as DNA damage, lipid peroxidation, and protein oxidation, potentially triggering apoptosis. ER stress, on the other hand, occurs when unfolded or misfolded proteins accumulate in the ER lumen, disrupting cellular homeostasis and activating various signaling pathways. Prolonged or severe ER stress can result in cell death via apoptosis [10,12].

At the molecular level, severe accumulation of reactive oxygen species triggers the Unfolded Protein Response (UPR) [13,14]. This process is initiated by the dissociation of the master chaperone GRP78 (BiP) from ER transmembrane sensors, leading to the activation of signaling branches including IRE1α and ATF6 [15]. While initially protective, unresolvable oxidative and ER stress transitions the UPR toward a pro-apoptotic state. This shift is primarily driven by the upregulation of the transcription factor CHOP (GADD153), which subsequently alters the balance of the Bcl-2 protein family [16]. By downregulating anti-apoptotic defenders like Bcl-2 and promoting pro-apoptotic executors such as Bax, the cell is irreversibly pushed toward mitochondrial membrane permeabilization and executioner Caspase-3-mediated apoptosis [17].

Tempol (4-hydroxy-2,2,6,6-tetramethylpiperidine-1-oxyl) is a nitroxide radical that exhibits antioxidant properties at low concentrations by scavenging free radicals and protecting cells from oxidative damage [18]. However, at high concentrations, Tempol can have pro-oxidant effects and increase ROS production in cells, potentially triggering apoptosis [19]. ML210 is a selective inhibitor that induces ferroptosis by inhibiting the glutathione peroxidase 4 (GPX4) enzyme. GPX4 is an important enzyme that protects cells against lipid peroxidation, and its inhibition can lead to damage to cell membranes and cell death [20].

Recent highly cited studies highlight that therapy-resistant melanomas exhibit a profound dependency on the GPX4 antioxidant axis, making the induction of ferroptosis a highly promising therapeutic vulnerability [21]. However, targeting a single cell death pathway often leads to compensatory resistance. Therefore, combining a targeted GPX4 inhibitor like ML210 with a redox-modulating agent that simultaneously floods the cell with ROS could theoretically overwhelm the tumor’s adaptive mechanisms [22]. By dismantling the lipid repair network while concurrently amplifying global oxidative and ER stress, such a combination strategy aims to trigger multiple, converging modalities of programmed cell death [23].

In this study, we aimed to understand the molecular mechanisms underlying the synergistic effects of the Tempol and ML210 combination in B16F10 murine melanoma cells and to evaluate its therapeutic potential. Our hypothesis is that the combination of Tempol and ML210 will synergistically induce cell death in B16F10 cells by increasing oxidative stress, triggering ER stress, and inducing apoptosis.

## 2. Results

### 2.1. Tempol and ML210 Dose-Dependently Inhibit B16F10 Cell Proliferation

**Figure 1:**
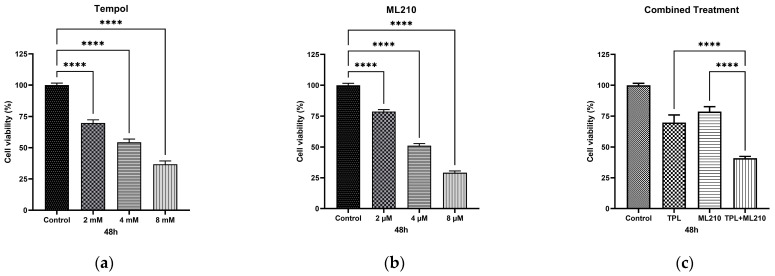
Cytotoxic effects of Tempol, ML210 and their combination on B16F10 melanoma cells. Cells were treated with indicated concentrations of (**a**) Tempol (2–8 mM), (**b**) ML210 (2–8 μM) and (**c**) the combination of 2 mM Tempol and 2 µM ML210 for 48 h. Cell viability was assessed by MTT assay. Data are presented as mean ± SEM (n = 6). Statistical significance was analyzed by one-way ANOVA followed by Dunnett’s multiple comparisons test (****: *p* < 0.0001).

To evaluate the cytotoxic effects of Tempol and ML210 individually, B16F10 melanoma cells were exposed to varying concentrations of each agent for 48 h. The MTT assay results revealed that both compounds significantly reduced cell viability in a dose-dependent manner compared to the control group (Figure 1). For Tempol, cell viability decreased to approximately 70%, 54%, and 37% at concentrations of 2 mM, 4 mM, and 8 mM, respectively (Figure 1a). Similarly, ML210 treatment resulted in viability rates of approximately 79%, 51%, and 29% at concentrations of 2 μM, 4 μM, and 8 μM, respectively (Figure 1b). Based on these cytotoxicity profiles, the 2 mM dose for Tempol and the 2 μM dose for ML210 were selected for subsequent combination experiments. These concentrations were chosen because they induced moderate cytotoxicity (sub-lethal), allowing for the potential observation of synergistic effects without causing excessive cell death initially. Furthermore, to confirm the efficacy of this co-treatment, we evaluated the cytotoxicity of the combination. As shown in Figure 1c, the combined treatment of 2 mM Tempol and 2 µM ML210 significantly reduced cell viability to approximately 41%, demonstrating a markedly enhanced cytotoxic effect compared to either monotherapy.

To formally quantify and validate the synergistic interaction between Tempol and ML210, a Bliss independence analysis was performed based on the MTT cell viability data. As detailed in Table 1, the observed effect of the combination treatment was significantly greater than the expected additive effect. The resulting positive Bliss score mathematically confirms the potent synergism of the Tempol and ML210 co-treatment in B16F10 cells.

### 2.2. Combination Treatment Upregulates ER Stress Markers

**Figure 2:**
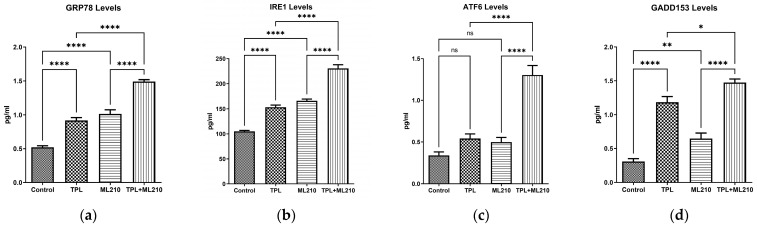
Effect of Tempol, ML210, and their combination on ER stress markers. The protein levels of (**a**) GRP78, (**b**) IRE1α, (**c**) ATF6, and (**d**) GADD153 were quantified by ELISA. Data are presented as mean ± SEM (n = 6). Statistical significance was analyzed by one-way ANOVA followed by Bonferroni’s multiple comparisons test (****: *p* < 0.0001, ** *p* < 0.01, *: *p* < 0.05: ns: not significant).

To investigate whether the combination treatment activates the ER stress response, the expression levels of GRP78, IRE1α, ATF6 and GADD153 (CHOP) were analyzed by ELISA. As shown in Figure 2, the regulation of these markers varied depending on the treatment protocol. For GRP78 and IRE1α, both Tempol and ML210 monotherapies significantly increased expression levels compared to the control group (*p* < 0.0001). However, the combination treatment induced a further and significant elevation in these proteins compared to the individual monotherapies (*p* < 0.0001), suggesting an enhanced stress response.

A distinct pattern was observed for ATF6. Neither Tempol nor ML210 alone caused a statistically significant change in ATF6 levels compared to the control (*p* > 0.05). In sharp contrast, the combination treatment triggered a robust and significant surge in ATF6 expression, which was significantly higher than both the control and single-agent groups (*p* < 0.0001).

Regarding the pro-apoptotic transcription factor GADD153 (CHOP), Tempol alone induced a strong upregulation (*p* < 0.0001), while ML210 caused a moderate but significant increase (*p* < 0.01) compared to the control. Consistent with other markers, the combination treatment resulted in the highest expression levels, significantly surpassing the effects of both Tempol (*p* < 0.05) and ML210 (*p* < 0.0001) alone.

### 2.3. Combination Treatment Regulates the Expression of Apoptotic Proteins

**Figure 3:**
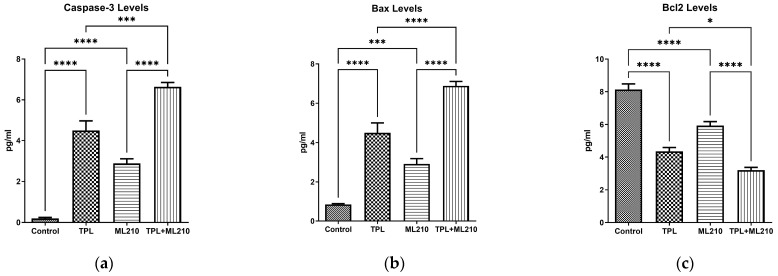
Effect of Tempol and ML210 on the expression of apoptotic proteins. The protein levels of (**a**) Cleaved Caspase-3, (**b**) Bax, and (**c**) Bcl-2 were determined by ELISA. Data are presented as mean ± SEM (n = 6). Statistical significance was analyzed by one-way ANOVA followed by Bonferroni’s multiple comparisons test (****: *p* < 0.0001, ***: *p* < 0.001, *: *p* < 0.05).

To determine whether the cell death induced by the combination treatment was mediated by apoptosis, the expression levels of key apoptotic regulators were examined. As presented in Figure 3, both Tempol and ML210 monotherapies significantly upregulated the expression of cleaved caspase-3 and Bax compared to the control group (*p* < 0.0001). Notably, the combination of Tempol and ML210 exerted a synergistic effect, resulting in significantly higher levels of these pro-apoptotic markers compared to either agent applied alone (*p* < 0.0001 for both markers against both monotherapies).

Conversely, the expression of the anti-apoptotic protein Bcl-2 showed a significant downregulation following treatment. While both Tempol (*p* < 0.0001) and ML210 (*p* < 0.0001) individually reduced Bcl-2 levels compared to the control, the combination treatment led to the most profound suppression of Bcl-2 expression. This reduction in the combination group was statistically significant compared to both the ML210-only group (*p* < 0.0001) and the Tempol-only group (*p* < 0.05).

### 2.4. Combination Treatment Shifts the Redox Balance

**Figure 4:**
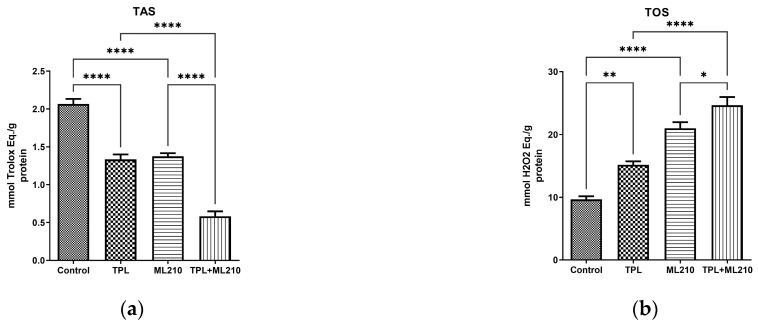
Effect of Tempol and ML210 on the global redox status of B16F10 cells. (**a**) Total Antioxidant Status (TAS) levels. (**b**) Total Oxidant Status (TOS) levels. Data are presented as mean ± SEM (n = 6). Statistical significance was analyzed by one-way ANOVA followed by Bonferroni’s multiple comparisons test (****: *p* < 0.0001, ** *p* < 0.01, *: *p* < 0.05).

To provide a comprehensive assessment of the cellular redox state, Total Antioxidant Status (TAS) and Total Oxidant Status (TOS) were quantified. As illustrated in Figure 4a, the TAS levels were significantly compromised by the treatments. Compared to the control group, both Tempol and ML210 monotherapies caused a significant reduction in antioxidant capacity (*p* < 0.0001). However, the combination treatment led to a near-total depletion of antioxidant reserves. This reduction was statistically significant when compared to both Tempol and ML210 applied alone (*p* < 0.0001).

Conversely, TOS levels exhibited a significant dose-dependent increase (Figure 4b). While the control group maintained a baseline oxidant status, Tempol treatment induced a significant increase (*p* < 0.01), and ML210 treatment resulted in a highly significant elevation (*p* < 0.0001) in oxidant levels. The combination treatment resulted in the highest recorded TOS values. This elevation was significantly superior to the effect of Tempol alone (*p* < 0.0001). Moreover, despite the high oxidant levels induced by ML210 monotherapy, the combination treatment still resulted in a statistically significant further increase compared to ML210 (*p* < 0.05).

### 2.5. Tempol and ML210 Combination Increases Intracellular H_2_O_2_ Levels

**Figure 5:**
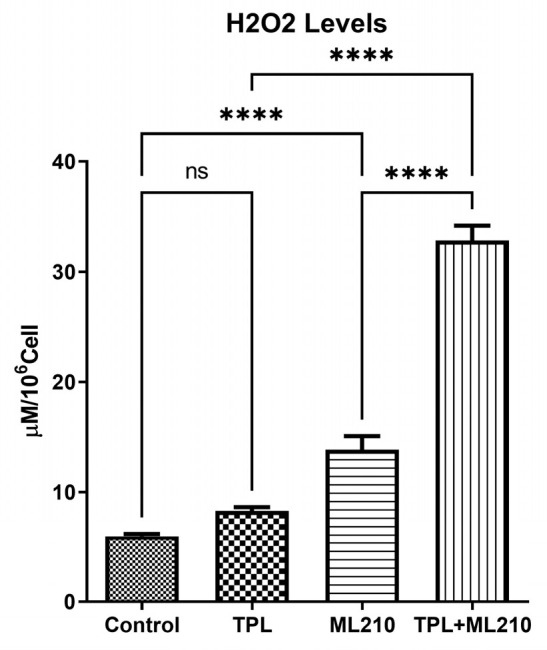
Effect of Tempol and ML210 combination on intracellular H_2_O_2_ levels. Data are presented as mean ± SEM (n = 6). Statistical significance was analyzed by one-way ANOVA followed by Bonferroni’s multiple comparisons test (****: *p* < 0.0001, ns: not significant).

To elucidate the mechanism underlying the cytotoxic effects, intracellular H_2_O_2_ levels were measured using a fluorescence-based assay. As shown in Figure 5, Tempol monotherapy resulted in a slight numerical increase in H_2_O_2_ levels compared to the control group (Mean: 8.3 vs. 5.98); however, this difference was not statistically significant (*p* > 0.05). In contrast, ML210 treatment significantly elevated H_2_O_2_ levels compared to the control (*p* < 0.0001). Notably, the combination of Tempol and ML210 led to a dramatic surge in H_2_O_2_ generation, reaching a mean value of 32.83. This increase was significantly higher than that of the individual monotherapies (*p* < 0.0001).

## 3. Discussion

This research highlights a promising and multifaceted approach to melanoma treatment by leveraging the synergistic effects of Tempol and ML210. Melanoma remains one of the most challenging skin cancers to treat due to its high metastatic potential and notorious resistance to conventional therapies [24]. Therefore, developing strategies that attack tumor cells through multiple mechanisms simultaneously could significantly enhance therapeutic efficacy and overcome resistance.

The core innovation in this study lies in targeting the redox balance within melanoma cells. Tempol, traditionally recognized for its antioxidant properties, exhibits pro-oxidant behavior at high concentrations, making it capable of elevating ROS levels in cancer cells [25]. However, our specific experimental data revealed a critical nuance regarding this mechanism. While Tempol (2 mM) monotherapy induced a numerical increase in intracellular H_2_O_2_ levels, this elevation was not statistically significant compared to the control, suggesting that the intrinsic antioxidant capacity of B16F10 cells could initially buffer this moderate oxidative pressure. Strikingly, the addition of ML210 dramatically altered this scenario. Since ML210 is a known GPX4 inhibitor, its addition likely impaired the cells’ ability to detoxify lipid peroxides, thereby sensitizing them to Tempol [26]. Consequently, the combination treatment triggered a massive, synergistic surge in H_2_O_2_ levels that neither agent could achieve individually.

When used together, Tempol and ML210 produce a potent synergistic effect, markedly amplifying oxidative stress beyond what either could achieve alone. This combined treatment significantly depletes the tumor cells’ antioxidant defenses, contributing to widespread cellular damage. This collapse of the cellular defense system was quantitatively confirmed by our TAS and TOS analyses. While ML210 acts as a specific inhibitor of GPX4, impairing lipid repair, our results revealed that the combination treatment led to a near-total depletion of the TAS, dropping significantly below the levels observed with monotherapies. Concurrently, the TOS reached its peak in the combination group, indicating an excessive accumulation of reactive species that the compromised defense system could not neutralize. This drastic shift in the redox balance—characterized by maximized oxidant burden and depleted antioxidant reserves—creates a severe oxidative imbalance, which likely serves as a primary driver for the subsequent activation of ER stress and apoptotic pathways [27]. The increase in oxidative stress not only directly damages cell membranes, DNA, and proteins but also activates stress response pathways such as ER stress, which is closely intertwined with oxidative damage [28].

ER stress, triggered by the accumulation of misfolded proteins in the endoplasmic reticulum, ultimately leads to cell death if unresolved [29]. In this study, the combination of Tempol and ML210 activated the Unfolded Protein Response (UPR) through multiple sensors. We observed a significant upregulation of the chaperone GRP78 (BiP) and the stress sensor IRE1α. Notably, the transcription factor ATF6 exhibited a distinct synergistic pattern; while monotherapies failed to induce significant changes, the combination treatment triggered a robust surge in ATF6 expression. This suggests that the dual treatment engages multiple branches of the UPR simultaneously. The unresolved stress ultimately led to the upregulation of the pro-apoptotic transcription factor CHOP (GADD153), illustrating that ER stress is a central component of the cell death mechanism here. Excessive ROS can disrupt ER function, impairing protein folding and thus exacerbating ER stress levels, which favor apoptosis through activation of downstream signaling pathways [30].

Mechanistically, the synergistic cell death observed in this study is strongly supported by the dysregulation of Bcl-2 family proteins, which serve as pivotal arbiters of the intrinsic apoptotic pathway [31]. Oxidative and ER stress are known to converge on mitochondria to initiate apoptosis [27]. In alignment with these findings, the combination therapy significantly downregulated the anti-apoptotic protein Bcl-2 while concurrently inducing a synergistic upregulation of pro-apoptotic Bax. This substantial alteration in the Bax/Bcl-2 ratio predisposes cells to mitochondrial outer membrane permeabilization, a decisive event in the apoptotic cascade [32]. Consequently, this cascade culminated in the robust activation of the executioner caspase-3, as evidenced by the significant accumulation of cleaved caspase-3 in the combination group. These findings indicate that Tempol and ML210 cotreatment not only depletes antioxidant defenses but also strongly promotes apoptotic signaling in B16F10 cells via the mitochondrial pathway.

Furthermore, the integration of intense oxidative damage and apoptotic signaling pathways—provides a comprehensive attack on melanoma cells. This multi-pronged approach has the potential to overcome the heterogeneous and adaptable nature of melanoma, which often develops resistance to therapies targeting only a single pathway [33]. For example, through mechanisms potentially involving GPX4 inhibition and oxidative imbalance, the strategy circumvents traditional resistance mechanisms associated with apoptosis; simultaneously, ER stress serves as an additional cell killing mechanism, pushing cells toward programmed death even if one pathway is compromised [10,34].

Clinically, these findings pave the way for designing combinatorial treatment protocols that leverage redox modulation, ferroptosis induction, and ER stress activation. Such strategies could be especially valuable in cases where tumors are resistant to chemotherapy, targeted agents, or immunotherapies. However, to translate these promising in vitro results into effective clinical treatments, further validation is necessary. We acknowledge that a limitation of this current study is the reliance on a single murine cell line (B16F10). Therefore, future studies must evaluate this combination in a diverse panel of human melanoma cell lines to confirm its broader translational relevance. Furthermore, extensive in vivo studies are required to rigorously evaluate factors such as optimal dosing, potential toxicities, pharmacokinetics, and long-term safety.

While our findings provide robust biochemical evidence for the molecular mechanisms triggered by the Tempol and ML210 combination, we acknowledge certain limitations in our study. Specifically, the activation of apoptosis was primarily evaluated through the expression of key apoptotic regulatory proteins (Bax, Bcl-2, and executioner Caspase-3) via quantitative ELISA. Although these are hallmark indicators of the apoptotic signaling cascade, future studies should incorporate functional confirmations such as flow cytometric Annexin V/PI staining. Furthermore, while ML210 is a recognized GPX4 inhibitor known to induce ferroptosis, our study focused on the resulting global redox imbalance (TAS/TOS and H_2_O_2_ accumulation) rather than directly measuring lipid peroxidation or performing rescue experiments with ferroptosis inhibitors. Therefore, while the severe oxidative crisis is evident, the definitive functional execution of ferroptosis in this specific combination model warrants further direct investigation.

Future research should also explore the potential for integrating these agents with existing therapies to enhance overall response rates and durability of treatment. There is also scope to investigate whether similar synergy can be observed in other cancer types characterized by high oxidative stress or resistance to conventional therapies. Additionally, developing targeted delivery systems could help minimize off-target effects and improve therapeutic indices.

In conclusion, this study demonstrates that the combination of Tempol and ML210 exhibits synergistic effects in B16F10 melanoma cells and may be a promising strategy in melanoma treatment. Combination treatment provides cell death by increasing oxidative stress, triggering ER stress, and promoting molecular changes consistent with apoptotic signaling. Future studies should evaluate the efficacy and safety of this combination in in vivo models. Additionally, it should be investigated how combination treatment affects the development of resistance in melanoma cells and in which patient subgroups it may be most effective. These findings emphasize the potential of combined treatment approaches to develop more effective and personalized treatment strategies in melanoma treatment.

## 4. Materials and Methods

### 4.1. Cell Culture and Maintenance

B16F10 murine melanoma cells were obtained from the American Type Culture Collection (ATCC, CRL-6475, Manassas, VA, USA) and maintained in Dulbecco’s Modified Eagle Medium (DMEM, Thermo Fischer, Waltham, MA, USA) enriched with 10% fetal bovine serum (FBS), 1% penicillin-streptomycin, and 2 mM L-glutamine. Cultures were kept in a humidified atmosphere containing 5% CO_2_ at 37 °C. Routine subculturing was performed, and cells in the logarithmic growth phase were selected for all experimental procedures.

### 4.2. Treatment Protocols

B16F10 cells were treated with different concentrations of Tempol (2 mM, 4 mM, 8 mM) and ML210 (2 μM, 4 μM, 8 μM) alone or in combination for 48 h. Based on the cell viability results from the MTT assay, the optimal doses were determined to be 2 mM for Tempol and 2 μM for ML210. Consequently, these specific concentrations were utilized for the combination treatments in all subsequent molecular analyses. The control group consisted of cells without any treatment.

### 4.3. Cell Viability Assay (MTT)

Cell viability was determined using the 3-(4,5-dimethylthiazol-2-yl)-2,5-diphenyltetrazolium bromide (MTT, Thermo Fischer, MA, USA) assay. Cells were seeded into 96-well plates and after exposure to the indicated treatments, MTT solution (5 mg/mL) was added to each well and incubated for 4 h [35]. Subsequently, formazan crystals were dissolved in dimethyl sulfoxide (DMSO, Thermo Fischer, MA, USA), and the absorbance was measured at 570 nm using a microplate reader (Rayto, RT-2100C, Shenzhen, China). Cell viability was expressed as a percentage relative to the untreated control group. The profound dose-dependent cytotoxicity observed at the highest concentrations of the tested agents served as functional validation of the assay’s capacity to detect cell death.

### 4.4. Sample Homogenization

Post-treatment (48 h), cells were harvested and spun down at 2000 rpm for 10 min at 4 °C. After discarding the medium, the pellets were washed twice with 5 mL PBS. Protein extraction was achieved by adding 250 μL of RIPA lysis buffer containing 2.5 μL of PMSF (200 mM), sodium vanadate (100 mM), and a protease inhibitor cocktail. The lysates were sonicated on ice and clarified by centrifugation at 10,000 rpm for 10 min at 4 °C. The resulting supernatants were collected for subsequent protein analysis [36].

### 4.5. Total Protein Quantification and Enzyme-Linked Immunosorbent Assay (ELISA)

Total protein levels were determined via the Bradford assay. A calibration curve was established using bovine serum albumin (BSA, Thermo Fischer, MA, USA) standards ranging from 1 to 10 μg/mL. For sample preparation, an aliquot of 10 μL from the homogenate was brought to a total volume of 100 μL using distilled water. Then, 1 mL of Bradford reagent was added to the sample. Upon thorough mixing, the optical density was quantified at 595 nm. Protein concentrations (μg/μL) were derived from the BSA standard curve using GraphPad Prism software (version 9; GraphPad Software, San Diego, CA, USA). All measurements were performed in sextuplicate. Following protein normalization, commercial ELISA kits (MyBioSource, San Diego, CA, USA) were utilized to evaluate the expression of apoptotic markers (cleaved caspase-3, Bax, Bcl-2) (cat #: MBS450868, MBS268932, MBS2881897) and ER stress proteins (GRP78, GADD153, IRE1α, ATF6) (cat #: MBS1601185, MBS1601186, MBS728814, MBS454285). The assays were conducted strictly according to the manufacturer’s protocols, and optical density was quantified using a microplate reader (Rayto, RT-2100C) [36].

### 4.6. Measurement of Total Antioxidant Status (TAS) and Total Oxidant Status (TOS)

TAS and TOS levels were quantified using specific kits obtained from Rel Assay Diagnostics (Gaziantep, Turkey), strictly following the manufacturer’s procedures included in the commercial kits. Standard Trolox and H_2_O_2_ solutions were run in parallel as internal positive controls and calibrators. Optical density was recorded at a wavelength of 660 nm via a spectrophotometer. Results were calculated as mmol Trolox Eq./g protein for TAS and μmol H_2_O_2_ Eq./g protein for TOS. Six independent replicates were analyzed for each group, with results expressed as mean ± standard deviation [36].

### 4.7. Measurement of Intracellular Hydrogen Peroxide (H_2_O_2_) Levels

Intracellular H_2_O_2_ levels were determined using an intracellular H_2_O_2_ assay kit (Abcam, cat # ab138874, Waltham, MA, USA). Known concentrations of standard H_2_O_2_ provided in the kit were utilized to generate a standard curve, serving as positive controls for the assay. Cells were treated as indicated, then processed according to the kit’s protocol, and fluorescence intensity was measured using a fluorescence microplate reader at an excitation wavelength of 490 nm and an emission wavelength of 520 nm. The absolute intracellular H_2_O_2_ concentrations were calculated based on the standard curve and are presented as µM per 10^6^ cells.

### 4.8. Statistical Analysis

All experiments were performed in six independent replicates. Data were expressed as mean ± standard error of the mean (SEM). Prior to parametric testing, the normal distribution of the data was verified using the Shapiro–Wilk test. Differences between groups were evaluated using one-way analysis of variance (ANOVA) followed by Bonferroni multiple comparisons test.

### 4.9. Synergy Analysis

The potential synergistic interaction between Tempol and ML210 was evaluated using the Bliss independence model. The expected additive effect of the combination was calculated based on the fractional responses of the individual agents. A positive Bliss score, indicating that the observed combination effect surpassed the mathematically expected additive effect, was considered indicative of synergism.

## Figures and Tables

**Table 1:** Bliss synergy analysis of the TPL (2 mM) and ML210 (2 µM) combination on cell viability.

	Observed Combination Viability (%)	Bliss Expected Viability (%)	∆ Bliss Score (Excess Inhibition)	Effect Type
TPL + ML210	40.79 ± 1.58	54.98 ± 2.28	14.18 ± 2.77	Strong Synergistic

Data are presented as mean % relative to the control ± SEM (n = 6). The expected fractional effect (inhibition) was calculated using the Bliss Independence formula (E_expected_ = E_TPL_ + E_ML210_ − E_TPL_ × E_ML210_). A ∆ Bliss score > 10 indicates strong synergy. Statistical significance of the difference between the observed and expected values was determined using a one-sample *t*-test (*p* < 0.001).

## Data Availability

The data presented in this study are available in Supplementary Material Data S1.

## Supplementary Materials

The following supporting information can be downloaded at: https://www.mdpi.com/article/10.3390/ijms27062675/s1.
